# Genome-Wide Identification of the Transient Receptor Potential Channel Family in Nile Tilapia and Expression Analysis in Response to Cold Stress

**DOI:** 10.3390/ani15243645

**Published:** 2025-12-18

**Authors:** Wanyue Deng, Yiqiong Wang, Qiong Niu, Qin Xu, Xuemei Wang, Yan Zheng, Deshou Wang, Ling Wei

**Affiliations:** Integrative Science Center of Germplasm Creation in Western China (Chongqing) Science City, Key Laboratory of Freshwater Fish Reproduction and Development (Ministry of Education), School of Life Sciences, Southwest University, Chongqing 400715, China

**Keywords:** Nile tilapia, TRP, gene duplication, expression profiling, cold sensing

## Abstract

Transient receptor potential (TRP) channels play critical roles in animals in sensing diverse stimuli, especially environmental temperature. The teleost fish Nile tilapia (*Oreochromis niloticus*) cannot tolerate cold temperatures. In this study, we identified a total of 32 *TRP* genes in the tilapia genome. Based on analyses of gene structure and phylogenetic relationship, all tilapia *TRP* genes could be classified into six subfamilies, namely, TRPA, TRPC, TRPM, TRPV, TRPP, and TRPML. Comparative analysis showed that three *TRP* subfamilies—TRPC, TRPM, and TRPML—underwent an expansion in tilapia and other teleost fishes following three or four rounds of whole-genome duplication. In addition, expression profiling revealed that a large number of *TRP* genes were expressed in at least one tissue in adult tilapia. Notably, compared with normal growth temperature (28 °C), cold stress (10 °C) altered the expression of several *TRPs* in multiple tissues in adult tilapia, especially upregulating *TRPC5* in the brain and *TRPM7* in the gill. Collectively, these findings provide new insight into the phylogeny of *TRP* genes in animals and lay the foundation for further investigation into the roles of TRP channels in cold sensitivity in tilapia.

## Simple Summary

The Nile tilapia (*Oreochromis niloticus*), an economically important farmed fish species, cannot survive in cold water temperatures, yet the underlying molecular mechanisms remain poorly understood. Transient receptor potential (TRP) channels play essential roles in the sensing of environmental temperature in animals. This study aimed to identify members of the TRP family in tilapia and investigate their response to cold stress. A genome-wide analysis predicted 32 *TRP* genes in the tilapia genome. We further characterized the sequence structure, chromosomal location, and molecular properties of the identified tilapia TRPs using bioinformatics tools. We also found that two *TRP* genes, namely, *TRPC5* and *TRPM7*, were upregulated in temperature-sensitive tissues of adult tilapia in response to cold temperatures, indicating that they are likely associated with cold sensing in tilapia. This work provides insight into potential roles of TRP channels in temperature sensing in tilapia.

## 1. Introduction

The transient receptor potential (TRP) family consists of integral membrane proteins that function as cation channels to sense and transduce a variety of stimuli in animals. The first TRP channel was identified in the fruit fly (*Drosophila melanogaster*) [1]. To date, more than 60 TRP channels have been identified in invertebrates and vertebrates [2]. For example, previous studies have reported 13 TRPs in the fruit fly [3], 17 in the nematode (*Caenorhabditis elegans*) and Zhikong scallop (*Chlamys farreri*) [4,5,6], 27 in the zebrafish (*Danio rerio*) [6], 28 in mouse (*Mus musculus*) and human (*Homo sapiens*) [4,7], and 66 in the pacific oyster (*Crassostrea gigas*) [8]. Structurally, TRP proteins contain different numbers of transmembrane helical segments [5,9,10]. In addition, several specific domains/motifs, such as N-terminal anchor repeat sequences (ARs) and C-terminus coiled helices, can be found in some TRP proteins [5]. Based on the differences in amino acid sequence and structure, the TRP family can be divided into nine subfamilies, namely, TRPA (ankyrin), TRPC (canonical), TRPM (melastatin), TRPML (mucolipin), TRPP (polycystin), TRPV (vanilloid), TRPN (nompC), TRPS (soromelastatin), and TRPVL (vanilloid-like); the first six subfamilies are present in mammals [3,5,9,11,12].

The TRP family plays important roles in various physiological processes, especially sensory reception in animals. Increasing evidence has shown that TRP proteins are involved in animals’ responses to different stimuli, such as light, temperature, sound, chemicals, and touch [5,13]. For instance, the first-identified TRP in the fruit fly can sense light stimuli [1,14]. Several TRP channels, including TRPAl, TRPC1, TRPV2, TRPV4, TRPM3, TRPP1, TRPP2, and TRPML3, are associated with mechanosensation [15]. Some TRPs, including TRPC2, TRPV1, TRPV4, TRPM5, and TRPM8, have been shown to act as chemosensors of sweet, bitter, or umami tastes [13,16]. Notably, previous reports have also demonstrated that a subset of TRPs, called thermoTRPs, can be activated by distinct external temperatures and thus confer temperature sensation [17,18]. In mouse, some thermoTRPs are sensitive to heat, such as TRPV1–4 and TRPM2–5, while other thermoTRPs respond to cold temperature, such as TRPC5 and TRPM8 [17,18]. Interestingly, TRPA1 acts as not only a cold sensor in nematodes and mammals but also a heat sensor in insects, amphibians, reptiles, and birds [18]. In addition to canonical sensory reception, several TRP channels also play essential roles in neurogenesis [19], reproduction [20], circadian rhythm [21], and pathogenesis [22,23].

The Nile tilapia (*Oreochromis niloticus*) is one of the most important farmed teleost fishes. A water temperature of 25–30 °C is optimal for its growth, survival, and reproduction [24,25,26], whereas a temperature less than 10–12 °C is lethal [27,28]. Thus, the Nile tilapia cannot be cultivated under low temperatures. In addition, heat treatments with high temperature above 32 °C to 36.5 °C can induce sex reversal of masculinization in tilapia [29,30,31]. Moreover, previous studies have reported transcriptome changes caused by a low temperature around 10 °C [32,33], or a high temperature of 36 °C [34,35,36]. However, the underlying mechanism of sensing and transducing temperature stress in tilapia, especially the roles of the TRP family members in thermosensation, remains poorly understood.

In the present study, with the availability of whole-genome sequences for tilapia [37], we conducted genome-wide identification and comparative analysis of the TRP family in tilapia. Through transcriptome analysis, in situ hybridization experiment, and RT-qPCR exanimation, the spatial expression of *TRP* genes and their responses to rearing temperature change were profiled. This study provides insight for further investigating the roles of the TRP family, especially in thermosensation in tilapia.

## 2. Materials and Methods

### 2.1. Animals

Nile tilapia fishes were cultivated in a circulating water system at 26 °C (unless otherwise indicated) under a 12 h light/12 h dark cycle in the key laboratory of freshwater fish resources and reproductive development (Ministry of Education) at Southwest University (Chongqing, China). All animal experiments were conducted in accordance with the guidelines approved by the Institutional Animal Care and Use Committee of Southwest University (No. IACUC-20181015-12, 15 October 2018).

### 2.2. Genome-Wide Prediction and Chromosomal Localization of the TRP Family

To comprehensively identify and compare the TRP family in the Nile tilapia and other representative animals, we performed BLAST (version 2.15.0) searches against their genome assemblies and annotated proteins deposited in the NCBI database using known TRP protein sequences from human, zebrafish, and fruit fly as the queries. In addition, we analyzed detailed sequence information of each tilapia *TRP* gene in the NCBI database to extract information about chromosomal localization.

### 2.3. Physicochemical Parameters and Functional Domain of TRP Proteins

Based on the amino acid sequence of annotated TRP proteins, the online Protparam program (https://web.expasy.org/protparam/; accessed on 10 May 2023) was used to estimate their molecular weight, theoretical isoelectric point (pI), instability index, and grand average of hydrophilicity (GRAVY). The online SOPMA program (https://npsa-prabi.ibcp.fr/cgi-bin/npsa_automat.pl?page=npsa_sopma.html; accessed on 16 May 2023) was used to predict the TRP proteins’ secondary structure, such as alpha helix, beta turn, and random coil. The BUSCA program (https://busca.biocomp.unibo.it/; accessed on 19 May 2023) was used to analyze their subcellular localization of TRP proteins. In addition, two online programs, CD-Search (https://www.ncbi.nlm.nih.gov/Structure/cdd/wrpsb.cgi; accessed on 23 May 2023) and SMART (version 9), were used to characterize the TRP proteins’ functional domains.

### 2.4. Phylogenetic and Syntenic Analyses of the TRP Genes

The complete amino acid sequences of TRP proteins from the Nile tilapia, zebrafish, spotted gar (*Lepisosteus oculatus*), and mouse were aligned using the MAFFT (L-INS-i) program (version 7.526) [38]. Based on the alignment results, a phylogenetic tree was constructed using the LG + Γ model with the Maximum Likelihood (ML) approach via the IQ-TREE software (version 2.4.0), with bootstrap values set to 1000 repetitions [39], and then visualized used the online iTOL program (version 6) [40]. In addition, we utilized newly updated genome assemblies for five representative animals deposited in the NCBI database to characterize the syntenic relationship for the *TRPM4* gene. The versions of the genome assembly are O_niloticus_UMD_NMBU for Nile tilapia, GRCh38.p14 for human, GRCm39 for mouse, GRCz12tu for zebrafish, and ASM223467v1 for medaka (*Oryzias latipes*).

### 2.5. Transcriptome-Based Expression Profiling of TRP Genes

Transcriptome data of ten tissues from adult tilapia reared under normal growth temperature, including blood, brain, eye, heart, kidney, liver, skeletal muscle, skin, testis, and ovary, were obtained from the NCBI database (accession number: PRJNA78915; https://www.ncbi.nlm.nih.gov/bioproject/?term=PRJNA78915; accessed on 4 October 2023) [37]; the data were used to profile the spatial expression of tilapia *TRP* genes. Transcriptome data of multiple tissues from adult tilapia reared under three different temperatures (28 °C, 18 °C, and 10 °C), downloaded from NCBI (accession number: GSE63727; https://www.ncbi.nlm.nih.gov/bioproject/?term=GSE63727; accessed on 16 October 2023) [41], were used to analyze the effect of low temperature on the expression of tilapia *TRP* genes. The Reads Per Kilobase per Million mapped reads (RPKM) values were used for expression profiling. As described previously [42], an RPKM threshold value < 1 was used to define a *TRP* gene as not detected in a tissue; in addition, the RPKM value was set as 1 ≤ RPKM ≤ 10 for low expression, 10 < RPKM ≤ 25 for moderate expression, and RPKM > 25 for high expression. The expression heatmap was drawn using the TBtools-II software (https://github.com/CJ-Chen/TBtools; accessed on 16 November 2023).

### 2.6. RT-PCR, RT-qPCR, and In Situ Hybridization Experiments

Semiquantitative RT-PCR experiments were conducted to evaluate the expression of *TRP* genes in various tissues of male adult tilapia at 180 days after hatching (dah) under 26 °C. Fifteen tissues—namely, skin, gill, blood, brain, pituitary, kidney, head kidney, spleen, intestine, liver, muscle, eye, heart, testis, and ovary—were dissected. Quantitative RT-PCR (RT-qPCR) analyses were performed to validate the expression changes in TRP genes following cold stress. As described in previous transcriptome analysis in tilapia [41], male adult tilapia at 180 dah were reared at 28 °C for two days. Then, the temperature was gradually decreased from 28 °C to 18 °C within 12 h at a rate of ~0.85 °C/h; after rearing for 12 h at 18 °C, the temperature was gradually dropped to 10 °C within 8.5 h, and the tilapia fishes were reared for 12 h at 10 °C. Three tissues—namely, brain, gill, and spleen—were separately collected from three individuals subjected to continuous rearing for 12 h at 28 °C, 18 °C, and 10 °C. Total RNA was extracted using the RNAiso Plus kit (Takara, Tokyo, Japan). A total of 1 μg RNA was reverse-transcribed into cDNA using M-MLV reverse transcriptase (Invitrogen, CA, USA). PCR was conducted as previously described [43]. Specifically, RT-qPCR was conducted using the SYBR1 Premix Ex TaqTM II kit (Takara, Tokyo, Japan) on the ABI-7500 PCR system (Applied Biosystems, Weiterstadt, Germany); the relative expression level was determined according to the 2^−ΔΔCT^ method. The tilapia *β-actin* gene was used as an internal control. Three samples were prepared for three biological replicates. The primers are listed in Supplementary Table S1.

For in situ hybridization, the cDNA fragment of tilapia *TRPC5* (including the 369 bp coding sequence and the 170 bp 3′ untranslated region) was cloned into the pGEM-T Easy vector. As described previously [44], this plasmid was employed to synthesize digoxygenin (DIG)-labeled *TRPC5* sense (control) and antisense (experimental) probes using the DIG RNA-labeling mix (Roche, Basel, Switzerland) and T7 RNA polymerase (Promega, Madison, WI, USA). The brain tissues from adult tilapia (180 dah) reared at 26 °C were fixed in 4% paraformaldehyde at 4 °C; the tissues were then dehydrated, embedded in paraffin wax and sectioned at 5 μm. The sections were deparaffinized, hydrated, and then hybridized with different probes overnight at 60 °C. After incubating the sections with a horseradish peroxidase (HRP)-conjugated anti-DIG antibody (1:1000; Roche, Basel, Switzerland), the expression signals were measured with the BCIP/NBT kit (Roche, Basel, Switzerland). Finally, the sections were visualized using an Olympus BX53 microscope (Olympus, Tokyo, Japan). The related primers are listed in Supplementary Table S1.

### 2.7. Statistical Analysis

Statistical values are presented as the mean ± SE for three independent biological replicates. Significant differences among more than two groups were determined using one-way ANOVA followed by Tukey’s test (with a threshold set at *p* < 0.05), and the results are indicated by different letters in the figures.

## 3. Results

### 3.1. In Silico Identification of TRP Genes in the Tilapia Genome

To characterize TRPs in the Nile tilapia, we performed a bidirectional best BLAST analysis to search against the tilapia genome sequence using known TRP protein sequences from multiple species (including human, zebrafish, and fruit fly) as queries, and then checked the existence of transmembrane segments in predicated TRP candidates. As a result, a total of 32 *TRP* genes were predicted from the tilapia genome (Table 1). These *TRP* genes could be classified into six subfamilies as follow: *TRPA1* in the TRPA subfamily, eight members (*TRPC1*, *TRPC2*, *TRPC4a*, *TRPC4b*, *TRPC5*, *TRPC6a*, *TRPC6b*, and *TRPC7*) in the TRPC subfamily, eleven members (*TRPM1a*, *TRPM1b*, *TRPM2*, *TRPM3*, *TRPM4a*, *TRPM4b1*, *TRPM4b2*, *TRPM4b3*, *TRPM5*, *TRPM6*, and *TRPM7*) in the TRPM subfamily, three members (*TRPV1*, *TRPV4*, and *TRPV6*) in the TRPV subfamily, four members (*TRPP1a*, *TRPP1b*, *TRPP2*, and *TRPP3*) in the TRPP subfamily, and five members (*TRPML1a*, *TRPML1b*, *TRPML2*, *TRPML3a*, and *TRPML3b*) in the TRPML subfamily.

We noted that each of six ancestral vertebrate *TRP* genes—namely, *TRPC4*, *TRPC6*, *TRPM1*, *TRPP1*, *TRPML1*, and *TRPML3*—had two copies, while *TRPM4* had four copies (Table 1), indicating that these *TRP* genes underwent a duplication in the Nile tilapia. Gene structure analysis revealed that the coding sequence (CDS) of the *TRPML2* gene was the shortest at 1515 bp in length, encoding 504 amino acid residues, and the CDS for *TRPP1a* was the longest at 13,989 bp, encoding 4662 amino acid residues (Table 1). The exon number of all tilapia *TRP* genes varied from 11 to 56 (Table 1).

**Figure 1:**
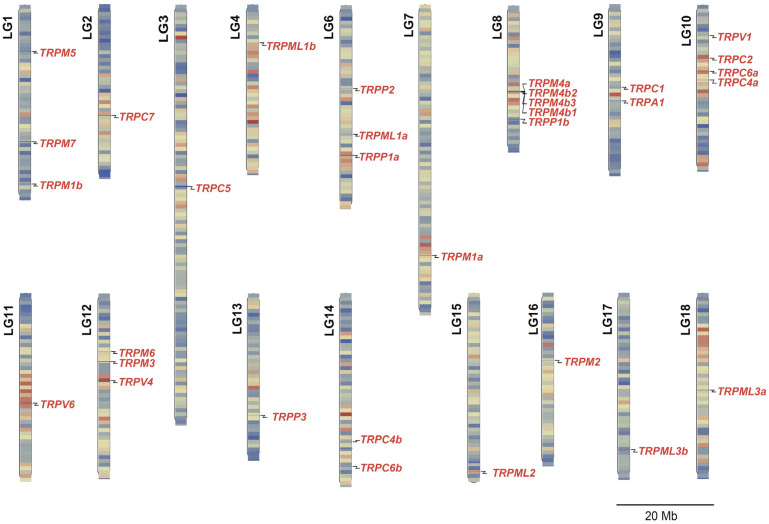
Chromosomal distribution of all identified *TRP* genes in the Nile tilapia. The precise position of each *TRP* gene on each linkage group (LG) is marked by a black line. The length of each LG is indicated as megabases (Mb). The duplicates of the *TRPM4* gene are tandemly clustered in LG8.

Further chromosomal mapping analysis showed that the 32 *TRP* genes were separately located on 17 linkage groups (LGs), presenting an uneven genomic distribution (Figure 1). In detail, only one *TRP* gene was located on LG2, LG3, LG4, LG7, LG11, LG13, LG15, LG16, LG17, or LG18. There were two or more *TRP* genes on LG1, LG6, LG8, LG9, LG10, LG12, or LG14. Notably, we observed that four copies of the *TRPM4* gene—namely, *TRPM4a*, *TRPM4b1*, *TRPM4b2*, and *TRPM4b3*—were located on LG8 and formed a gene cluster (Figure 1).

### 3.2. Molecular Properties of Tilapia TRP Proteins

Next, we in silico analyzed the molecular properties of the annotated protein sequences for all identified tilapia *TRP* genes using the Protparam programs available on the ExPASy website. First, as shown in Table 1, the predicted molecular weight of the 32 tilapia TRP proteins ranged from 58.58 kDa (TRPML2) to 515.83 kDa (TRPP1a). Second, the theoretical isoelectric point (pI) for all tilapia TRP proteins varied from 5.27 (TRPV4) to 8.53 (TRPP1a); among the 32 tilapia TRP proteins, 22 members were acidic (pI < 7) while the rest were alkaline (pI > 7) (Table 1). Third, regarding the instability coefficient described previously [45], we observed that seven tilapia TRP proteins—namely, TRPM5, TRPP3, TRPML1a, TRPML1b, TRPML2, TRPML3a, and TRPML3b—had an instability coefficient lower than 40 (Table 1), indicating that these TRP proteins were stable in vitro. Finally, regarding the grand average of hydropathicity (GRAVY), which is an index of a protein’s hydrophobicity [46,47], we found that most tilapia TRP proteins had a negative GRAVY value (Supplementary Table S2), indicating that they might be hydrophilic proteins. In contrast, eight TRP proteins—namely, TRPA1, TRPV6, TRPP1b, TRPML1a, TRPML1b, TRPML2, TRPML3a, and TRPML3b—had a positive GRAVY value (Supplementary Table S2), suggesting that these TRPs were hydrophobic.

We also predicted the secondary structure and subcellular localization of the tilapia TRP proteins. The results showed that the tilapia TRP proteins were composed of alpha helix ranging from 23.25 to 59.81%, beta turn at 2.52–7.87%, and random coil at 28.57–44.89% (Supplementary Table S2). In addition, regarding subcellular localization, our analysis revealed that eighteen TRP proteins—namely, TRPC2, TRPC5, TRPM2, TRPM4a, TRPM4b1, TRPM4b2, TRPM4b3, TRPM5, TRPM6, TRPM7, TRPV1, TRPP1a, TRPP1b, TRPP2, TRPP3, TRPML1a, TRPML3a, and TRPML3b—were localized on the plasma membrane (Supplementary Table S2). Fourteen TRP proteins—namely, TRPA1, TRPC1, TRPC4a, TRPC4b, TRPC6a, TRPC6b, TRPC7, TRPM1a, TRPM1b, TRPM3, TRPV4, TRPV6, TRPML1b, and TRPML2—were localized on the organelle membrane (Supplementary Table S2).

**Figure 2:**
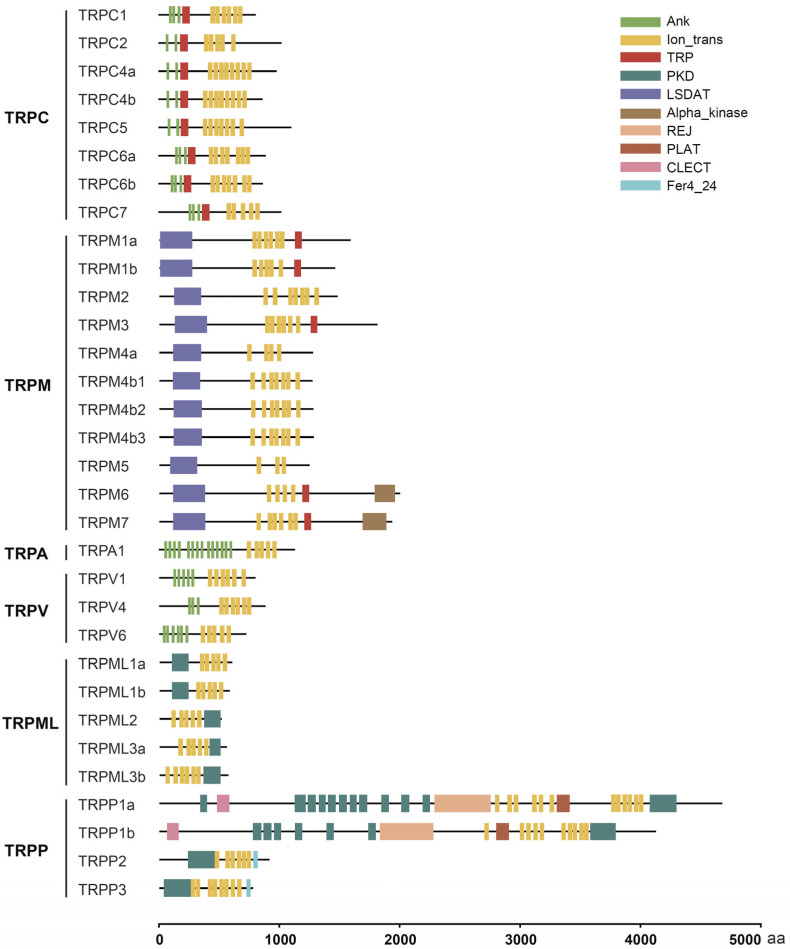
Functional domains of tilapia TRP proteins. The conserved transmembrane domain (lon_trans) and other types of functional domains are marked with different boxes and colors. The scale at the bottom denotes the length of each protein in terms of number of amino acid residues. aa, amino acid.

We further annotated the functional domains of these tilapia TRP proteins using the Conserved Domain Search Service (CD-Search) available on the NCBI database and the online SMART program. The results showed that all tilapia TRP proteins contained canonical transmembrane segments (lon_trans) at different numbers (Figure 2). In addition, we also observed several special domains in some TRP proteins. For example, the TRPC subfamily and five members of the TRPM subfamily contained a TRP domain with 23–25 amino acid residues (Figure 2). The N-terminus of most members of the TRPC, TRPA, and TRPV subfamilies contained anchor protein repeat (ANK) domains that mediate cytoskeleton anchoring or protein interactions (Figure 2). All members of the TRPM subfamily had an LSDAT domain that is necessary for TRP channels to recognize specific ligands; notably, two members of the TRPM subfamily—namely, TRPM6 and TRPM7—contained an Alpha_kinase domain that is involved in protein phosphorylation (Figure 2). All members of the TRPML and TRPP subfamilies contained different numbers of the polycystic kidney disease (PKD) domain (Figure 2).

### 3.3. Phylogenetic and Syntenic Relationships of TRPs in the Nile Tilapia and Other Animals

We next investigated the phylogenetic relationships of the *TRP* genes between the Nile tilapia and other representative animals. For a comprehensive comparison, we updated the number of TRP family members in several animals. Among teleost fishes, we identified 60 *TRP* genes in the common carp (*Cyprinus carpio*), 58 in rainbow trout (*Oncorhynchus mykiss*), 34 in channel catfish (*Ictalurus punctatus*), 33 in medaka, and 27 in the spotted gar. Compared with previous reports [6,48,49], the number of *TRP* genes was updated to 38 for the zebrafish, with the addition of two members, namely, *TRPM4b3* and *TRPP3* (Table 2 and Supplementary Table S3). Moreover, we newly identified a *TRP* gene, *TRPP5*, in mouse and human, therefore updating the number of their *TRP* genes to 29 (Table 2 and Supplementary Table S3).

Comparatively, we observed that the TRPN subfamily contained one copy in all analyzed animals. Notably, compared with the fruit fly as an invertebrate model, members of five *TRP* subfamilies—namely, TRPC, TRPM, TRPV, TRPP, and TRPML—underwent an obvious expansion in vertebrates (Table 2 and Supplementary Table S3). Among teleost fishes, compared with the spotted gar, an ancient fish lineage that radiated from the teleost lineage before the teleost-specific whole-genome duplication (WGD) and generally used as an outgroup for phylogenetic analysis [50,51], some members of three TRP subfamilies—namely, TRPC, TRPM, and TRPML—underwent duplication in most species of teleost fishes with three (3R) or four rounds (4R) of WGD and generated two or more copies, leading to an expansion (Table 2 and Supplementary Table S3).

**Figure 3:**
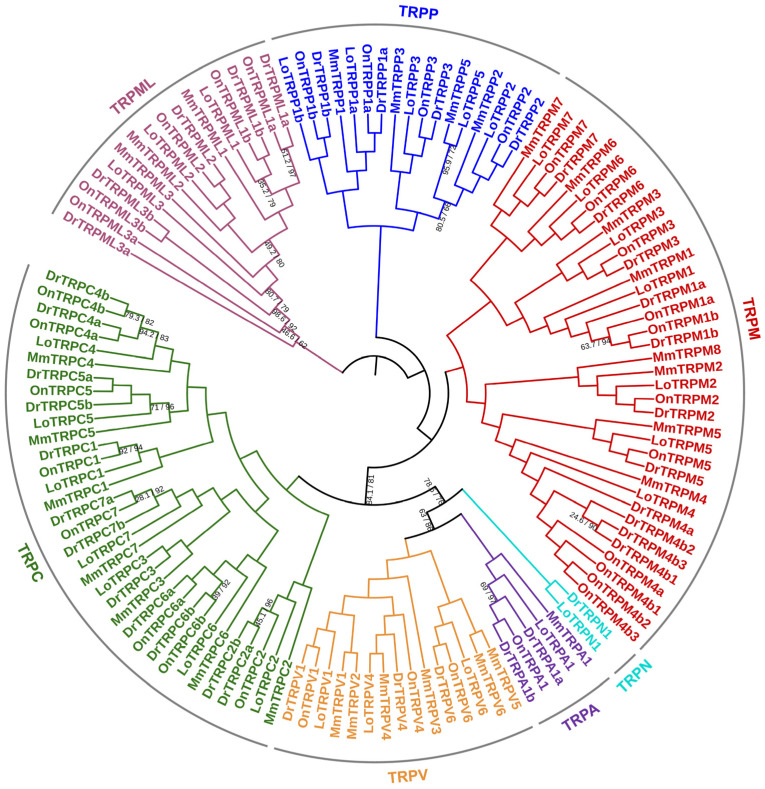
Phylogenetic tree of the TRP families identified in the Nile tilapia and other representative animals. The amino acid sequences of the TRP proteins identified in the Nile tilapia and three other animal species were aligned using the MAFFT (L-INS-i) program (version 7.526). Based on the alignment results, a phylogenetic tree of the TRP families in these animals was constructed using the LG + Γ model with the Maximum Likelihood (ML) approach via the IQ-TREE software (version 2.4.0), with bootstrap values set to 1000 repetitions. This phylogenetic tree was visualized using the online iTOL program (version 6). All nodes are supported by UFBoot values ≥ 95% and SH-aLRT values ≥ 80%, unless otherwise noted. The branch for each TRP subfamily is indicated by different colors. On, *Oreochromis niloticus* (Nile tilapia); Dr, *Danio rerio* (zebrafish); Mm, *Mus musculus* (mouse); Lo, *Lepisosteus oculatus* (spotted gar).

We built a phylogenetic tree of the TRP families in the Nile tilapia and other representative animals, including the spotted gar, zebrafish, and mouse, based on the amino acid sequences of the identified TRP proteins. The results showed that the TRP proteins from these four animals were clearly grouped into seven groups: TRPA, TRPC, TRPM, TRPN, TRPV, TRPP, and TRPML (Figure 3). In addition, we noted that the orthologs of each *TRP* gene identified in the Nile tilapia and other representative animals were well grouped together, indicating that these *TRP* genes originated before the radiation of these animals. Interestingly, among the duplicates (*a* and *b*) of several *TRP* genes identified in the Nile tilapia and zebrafish, including *TRPM1*, *TRPC4*, *TRPC6*, *TRPP1*, *TRPML1*, and *TRPML3*, all orthologous pairs—namely, *a* or *b*—were grouped together (Figure 3), indicating the duplication of these *TRP* genes occurred before the radiation of these two fish species. However, regarding the duplicates of *TRPM4*, all paralogous pairs—namely, *a* and *b*—identified in the Nile tilapia or zebrafish were grouped together in a species-dependent manner; the same was observed for the duplicates of *TRPM4b* (Figure 3). This observation indicated that the duplication of *TRPM4* occurred after the radiation of these two fish species.

**Figure 4:**
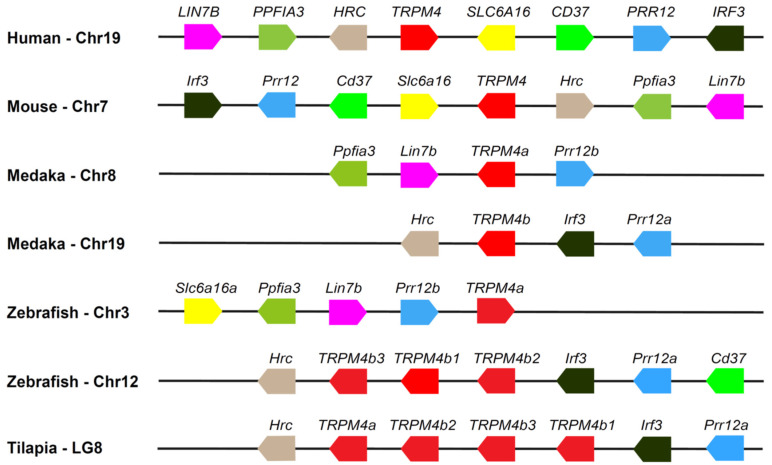
Syntenic map of the *TRPM4* gene in the Nile tilapia and other representative animals. The order of the *TRPM4* gene, its duplicates, and its adjacent genes are presented. The numbers of the chromosomes (Chr) or linkage groups (LG) are indicated following the species name. Arrow boxes with the same color represent the same gene or the duplicates of an ancestral gene. The *TRPM4* gene and its duplicates are denoted by red arrow boxes. The direction of each arrow box indicates the transcriptional direction of the corresponding gene. The length of the segments in the figure does not represent actual genetic distance.

Given that multiple copies of the *TRPM4* gene were tandemly distributed in LG8 in the Nile tilapia (Figure 1), we investigated the syntenic relationship of the *TRPM4* gene between the Nile tilapia and other vertebrates. The results showed that the tandem distribution of three copies of the *TRPM4b* gene also occurred on chromosome 12 in the zebrafish (Figure 4). In addition, we found that although the duplicates *a* and *b* of the *TRPM4* gene in the zebrafish and medaka were separately located on two chromosomes, *TRPM4* had the same neighboring genes, mainly *Prr12*, *Irf3*, and *Hrc* (Figure 4). These data further indicate that *TRPM4* is evolutionarily conserved in teleost fishes and other vertebrates, and its duplication in teleost fishes occurred following 3R or 4R WGD.

### 3.4. Spatial Expression Profile of TRP Genes in the Nile Tilapia

**Figure 5:**
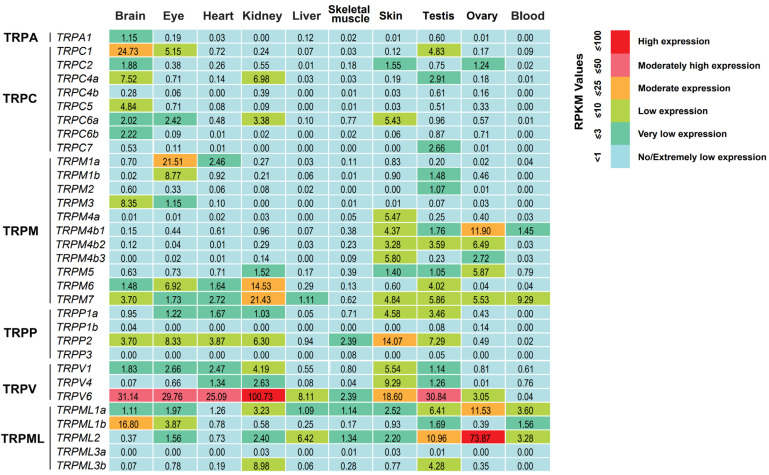
Transcriptome-based expression profiling of *TRP* genes in multiple tissues in adult tilapia reared under normal growth temperature. The transcriptome data were obtained from the SRA database in NCBI (accession number: PRJNA78915). The expression levels of these *TRP* genes were measured based on the RPKM values. The number within each box indicates the RPKM value.

To understand the potential functions of tilapia TRPs, we sought to profile the expression of the 32 *TRP* genes in multiple tissues in adult tilapia reared under normal growth temperature using public transcriptome data (NCBI accession number: PRJNA78915) [37]. Based on the criterion of RPKM (reads per kilobase per million mapped reads) values ≥ 1 for expression level, we found that except for *TRPC4b*, *TRPP1b*, *TRPP3*, and *TRPML3a*, the other 28 *TRP* genes could be transcriptionally detected in at least one tissue of adult tilapia (Figure 5). The numbers of *TRP* genes with detectable expression were 19 in the testis, 15 in the brain and skin, 14 in the eye, 13 in the kidney, 9 in the ovary, 8 in the heart, 5 in blood, 4 in the liver, and 4 in skeletal muscle.

**Figure 6:**
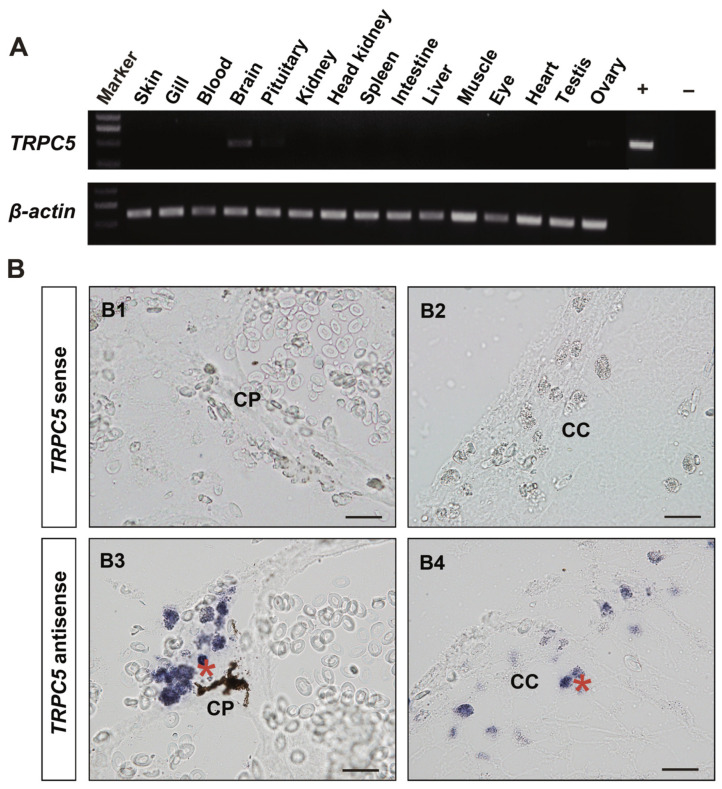
RT-PCR and in situ hybridization analyses of *TRPC5* expression in male adult tilapia (180 dah) reared at 26 °C. (**A**) RT-PCR analysis of *TRPC5* expression in different tissues in adult tilapia. The *β-actin* gene was used as the internal reference gene. +, positive control; −, negative control. (**B**) In situ hybridization analysis of *TRPC5* expression in the brain of adult tilapia. DIG-labeled *TRPC5* sense (control, **B1**,**B2**) and antisense (experimental, **B3**,**B4**) probes were used. The asterisk in red color indicates positive signal for *TRPC5* expression. CP, choroid plexus (**B1**,**B3**); CC, cerebral cortex (**B2**,**B4**). Scale bar: 50 μm.

We also observed that two tilapia *TRP* genes showed high expression with an RPKM value over 25, including *TRPV6* in the brain, eye, heart, kidney, and testis and *TRPML2* in the ovary (Figure 5). Ten *TRP* genes had moderate expressions (with an RPKM value greater than 10 but less than 25) in different tissues: *TRPC1* and *TRPML1b* in the brain, *TRPP2* and *TRPV6* in the skin, *TRPM6* and *TRPM7* in the kidney, *TRPM4b1* and *TRPML1a* in the ovary, *TRPML2* in the testis, and *TRPM1a* in the eye. Notably, six *TRP* genes exhibited tissue-specific expressions: *TRPC5*, *TRPC6b*, and *TRPA1* in the brain, *TRPM4a* in the skin, and *TRPC7* and *TRPM2* in the testis. Moreover, given that the brain-specific expression of *TRPC5* observed in the transcriptome analysis, we further performed RT-PCR and in situ hybridization experiments to validate *TRPC5* expression in adult tilapia. RT-PCR analysis showed that *TRPC5* was specifically expressed in the brain (Figure 6A), a result that was consistent with the transcriptome data. In situ hybridization analysis revealed that compared to the negative control with the sense RNA probe, *TRPC5* expression signal could be detected in the secretory cells of the cerebral choroid plexus and cerebral cortex (Figure 6B).

### 3.5. Expression Change in Tilapia TRP Genes Under Low Temperature Stress

**Figure 7:**
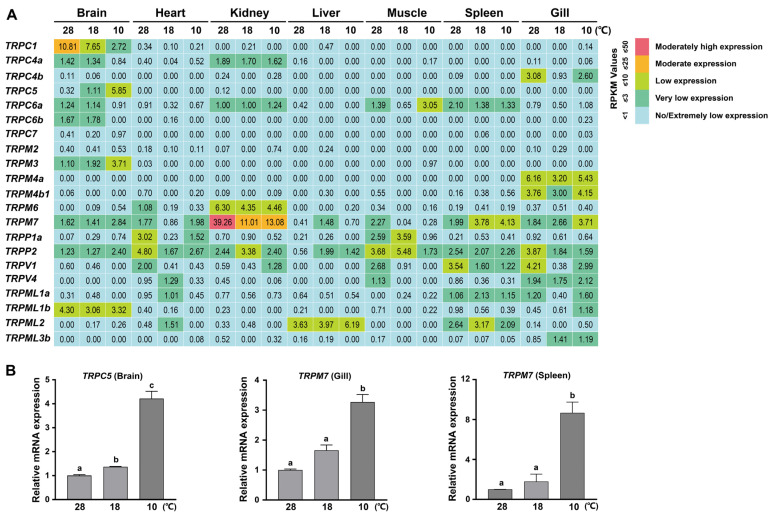
Low temperature-induced expression changes in TRP genes in adult tilapia. (**A**) Transcriptome-based expression profiling of the *TRP* genes in adult tilapia in response to low temperature. The transcriptome data of *TRP* genes in multiple tissues of adult tilapia (180 dah) subjected to different temperatures (28 °C, 18 °C, and 10 °C) were obtained from NCBI (accession number: GSE63727). The expression levels of *TRP* genes were measured based on the RPKM values. (**B**) RT-qPCR validation of the upregulated expressions of several *TRP* genes in several tissues in male adult tilapia (180 dah) by low temperatures (18 °C and 10 °C). Different letters on the bar indicate significant differences at *p* < 0.05 and the same letter indicates no significant difference.

A number of TRP proteins have been shown to play important roles in temperature sensation in animals [17,18]. Therefore, we further explored the expression change in the 32 *TRP* genes in several tissues in adult tilapia under low temperature stress. Based on the public transcriptome data of adult tilapia (180 dah) subjected to three different temperatures for 12 h, namely, 28 °C for suitable growth and low temperatures of 18 °C and 10 °C (NCBI accession number: GSE63727) [41], we found that several *TRP* genes presented an obvious expression change in different tissues following the low temperature treatment (Figure 7A). Specifically, five *TRP* genes were upregulated in response to cold stress, including *TRPC5*, *TRPM3*, *TRPM7*, and *TRPP2* in the brain, *TRPM7* in the gill and spleen, and *TRPML2* in the liver (Figure 7A). In contrast, several *TRP* genes presented a transcriptional downregulation following the low temperature treatment, including *TRPC1* in the brain, *TRPP2* in the gill, *TRPV1* in the spleen, and *TRPM7* in the kidney and muscle (Figure 7A). We further conducted RT-qPCR experiments in male adult tilapia (180 dah) and confirmed the upregulation of *TRPC5* expression in the brain and *TRPM7* expression in both the gill and spleen following cold stress (10 °C) compared with normal temperature (28 °C) (Figure 7B). Collectively, our data suggest that a subset of *TRP* genes are transcriptionally regulated by low temperature and may be involved in temperature-dependent physiological processes in different tissues in adult tilapia.

## 4. Discussion

TRP proteins contain specific transmembrane domains and are exclusively discovered in animals to date. The TRP family has been shown to be involved in various biological processes, especially sensing and transducing diverse stimuli such as light, temperature, sound, chemicals, and touch [5,13]. In this study, we predicted 32 *TRP* genes in the genome of the Nile tilapia. Compared to the ancient fish spotted gar with 2R WGD, three TRP subfamilies—namely, TRPC, TRPM, and TRPML—underwent an expansion in the Nile tilapia and other teleost fishes with 3R or 4R WGD. Expression profiling showed that a subset of tilapia *TRP* genes is transcriptionally modulated by low temperature. Our data provide new insights into the evolutionary divergence of the TRP family during teleost radiation and its potential function in the thermosensation of tilapia.

Among teleost fishes, genome-wide identification of the TRP family has been firstly reported in the zebrafish [52]. In this study, we not only identified *TRP* genes in the genomes of five teleost fish species, including the Nile tilapia, common carp, rainbow trout, channel catfish, and spotted gar, but also annotated an additional two *TRP* genes in the zebrafish genome. Our analysis revealed two interesting findings about the evolution of the TRP families in teleost fishes. First, six TRP subfamilies—namely, TRPA, TRPC, TRPM, TRPML, TRPP, and TRPV—exist in all teleost fish species analyzed in our study. TRPN (also called NOMPC) exists in invertebrates and some fish species (e.g., zebrafish, common carp, and rainbow trout) with a single copy, but it is not present in mammals and other fish species (e.g., tilapia and medaka). TRPN/NOMPC has been extensively studied in the fruit fly, functioning as a mechanosensitive channel to sense sound, touch, and proprioception [53,54,55,56,57,58,59]. In the zebrafish, TRPN/NOMPC mediates the mechanotransduction of sensory hair cells [60]. The loss of the *TRPN* gene in the Nile tilapia and medaka suggests that the mechanosensation in these fish species may be modulated by other TRP proteins. Second, compared to some vertebrates with 2R WGD (including mammals and the teleost fish spotted gar), several TRP subfamilies, including TRPM, TRPC, and TRPML, underwent an expansion in the Nile tilapia and other teleost fishes with 3R or 4R WGD. Our data suggest that the expansion of these TRP subfamilies is due to gene duplication events during WGD. In fact, WGD-derived expansion has been characterized for other gene families in teleost fishes [61,62,63]. Given that TRP proteins mainly function to sense diverse stimuli, especially environmental temperature [5,13], the expansion of the TRP subfamilies in the Nile tilapia and other teleost fishes is most likely linked to thermosensory adaptation during their evolution.

Increasing evidence has demonstrated that several TRP channels (ThermoTRPs) have the conserved function of facilitating the thermosensation of animals under temperature stimuli, including cold-sensitive ThermoTRPs, such as TRPC5 and TRPM8, and heat-sensitive ThermoTRPs, such as TRPV1 and TRPM2 [5,13,18,64,65]. Notably, TRPA1 has been shown to sense cold stress in mouse but respond to heat stimuli in insects, chicken, and lizard [66,67], uncovering a functional difference in thermosensation for TRPA1 among different animal species. In fishes, zebrafish TRPV1 and medaka TRPA1 function as heat sensors [68,69]. The Nile tilapia is sensitive to cold stress and cannot survive at water temperatures lower than 10 °C [27,28]. In this study, we found that in adult tilapia reared under normal temperature, *TRPC1*, *TRPV6*, and *TRPML1b* had high expression in the brain and/or skin, and several *TRPs* exhibited tissue-specific expression, including *TRPC5*, *TRPC6b*, and *TRPA1* in the brain and *TRPM4a* in the skin. Since the brain and skin are two temperature-sensitive tissues in the Nile tilapia, our observations indicate that these TRPs may directly correlate with the sensing of temperature stimuli in this fish species. Furthermore, transcriptome profiling and RT-qPCR examination further uncovered that compared with a suitable temperature of 28 °C, low temperatures (18 °C and 10 °C) upregulated the expression of *TRPC5* in the brain and *TRPM7* in the gill (a temperature-sensitive tissue). A previous report has demonstrated that TRPC5 is a cold sensor in mouse [70]. Thus, our finding regarding the cold-upregulated *TRPC5* expression in the brain suggests that TRPC5 may mediate cold sensitivity in the Nile tilapia. In future work, we will perform gain- and loss-of-function analyses to comprehensively investigate the roles of these TRPs in the thermosensation of the Nile tilapia, especially in cold sensing.

## 5. Conclusions

TRP channels play diverse physiological roles in animals in response to extracellular and intracellular stimuli, especially temperature sensing. In this study, we identified 32 TRP genes in the Nile tilapia, a cold-sensitive teleost fish. Compared to the ancient fish spotted gar with 2R WGD, three subfamilies—namely, TRPC, TRPM, and TRPML—have undergone gene expansion in the Nile tilapia and other teleost fishes with 3R or 4R WGD. A subset of *TRP* genes presented upregulated or downregulated expressions in different tissues in adult tilapia subjected to cold stress. Our data provide novel insights into the evolution of the TRP family in animals and important clues for further deciphering the roles of TRPs in thermosensation in tilapia. In the future, it may be worthwhile to precisely edit cold-responsive *TRP* genes to improve cold tolerance in tilapia farming.

## Figures and Tables

**Table 1:** Inventory of *TRP* genes in the tilapia genome.

Subfamily	Gene Name	Gene ID (NCBI)	LG *	Genome Position	CDS (bp)	Exon Number	Amino Acid Residues	Molecular Weight (KDa)	Theoretical pI
TRPA	*TRPA1*	100701720	9	20,077,700–20,120,065	3357	29	1118	124.58	6.59
TRPC	*TRPC1*	100704317	9	17,239,789–17,255,310	2388	13	795	91.84	8.49
	*TRPC2*	100708475	10	11,048,099–1,1056,756	3033	13	1010	115.54	5.52
	*TRPC4a*	100695568	10	15,614,154–15,643,024	2907	16	968	110.43	5.93
	*TRPC4b*	100692457	14	30,884,234–30,898,079	2556	14	851	98.26	6.28
	*TRPC5*	100707123	3	37,697,306–37,891,529	3276	18	1091	123	5.86
	*TRPC6a*	100706122	10	13,809,430–13,821,918	2640	14	879	98.96	6.01
	*TRPC6b*	100698870	14	32,251,012–32,258,627	2562	11	853	98.34	6.35
	*TRPC7*	100711831	2	23,091,431–23,148,709	3027	12	1008	115.05	6.31
TRPM	*TRPM1a*	100701437	7	52,174,974–52,194,948	4731	27	1576	179.27	8.33
	*TRPM1b*	106097084	1	37,227,995–37,250,918	4341	28	1446	164.35	6.78
	*TRPM2*	100711841	16	14,032,582–14,060,973	4407	34	1468	166.82	8.05
	*TRPM3*	100708457	12	14,069,166–14,184,539	5400	27	1799	202.76	6.98
	*TRPM4a*	102075974	8	17,836,154–17,860,845	3792	28	1263	143.4	6.33
	*TRPM4b1*	100703227	8	17,976,607–17,993,303	3783	28	1260	143.34	6.14
	*TRPM4b2*	109194528	8	17,861,294–17,897,107	3804	28	1267	144.37	6.44
	*TRPM4b3*	109203256	8	17,949,589–17,972,484	3813	29	1270	144.58	6.67
	*TRPM5*	100697354	1	9,639,846–9,658,173	3705	26	1234	140.23	5.9
	*TRPM6*	100711567	12	12,043,025–12,063,469	5961	40	1986	224.49	5.92
	*TRPM7*	109194405	1	28,439,676–28,540,542	5766	40	1921	217.36	8.19
TRPV	*TRPV1*	100701681	10	6,349,926–6,367,860	2361	17	786	91.45	6.34
	*TRPV4*	100708918	12	18,083,119–18,096,328	2613	17	870	98.39	5.27
	*TRPV6*	100696021	11	22,860,341–22,870,407	2136	17	711	80.67	5.93
TRPP	*TRPP1a*	100695955	6	31,187,112–31,258,030	13989	56	4662	515.83	8.53
	*TRPP1b*	100694735	8	23,583,712–23,615,821	12342	42	4113	454.13	7.99
	*TRPP2*	100701939	6	17,288,188–17,299,477	2706	15	901	102.4	8.48
	*TRPP3*	100701261	13	25,347,466–25,353,907	2298	15	765	87.86	6.28
TRPML	*TRPML1a*	100697794	6	26,836,158–26,853,964	1776	14	591	67.49	6.16
	*TRPML1b*	100708610	4	7,944,215–7,957,642	1716	13	571	64.23	7.47
	*TRPML2*	100697896	15	37,070,012–37,083,599	1515	14	504	58.58	5.65
	*TRPML3a*	100693840	18	20,178,984–20,184,700	1641	13	546	63.77	7.20
	*TRPML3b*	100694709	17	32,537,438–32,545,793	1680	15	559	65.53	7.17

* LG, linkage group.

**Table 2:** Number of TRP family members in the Nile tilapia and other animals.

Animals	WGD	TRPA	TRPC	TRPM	TRPN	TRPV	TRPP	TRPML	Total
Fruit fly	1R	4	3	1	1	2	1	1	13
Mouse	2R	1	7	8		6	4	3	29
Human	2R	1	7	8		6	4	3	29
Spotted gar	2R	1	7	7	1	3	5	3	27
Nile tilapia	3R	1	8	11		3	4	5	32
Medaka	3R	1	8	10		4	5	5	33
Zebrafish	3R	2	12	11	1	3	4	5	38
Channel catfish	3R	2	10	8	1	4	4	5	34
Common carp	4R	3	20	16	1	7	6	7	60
Rainbow trout	4R	4	17	19	1	6	4	7	58

## Data Availability

The transcriptome data used in this study were obtained from the NCBI database (accession number: PRJNA78915 and GSE63727) (https://www.ncbi.nlm.nih.gov/bioproject/?term=PRJNA78915 and https://www.ncbi.nlm.nih.gov/bioproject/?term=GSE63727; accessed on 16 October 2023).

## Supplementary Materials

The following supporting information can be downloaded at https://www.mdpi.com/article/10.3390/ani15243645/s1. Supplementary Table S1: Primer sequences used in this study; Supplementary Table S2: Molecular characteristics of TRP proteins in the Nile tilapia; Supplementary Table S3: Inventory of the TRP families in several representative animals. Reference [71] is cited in the Supplementary Materials.
