# Supplementary material for: Genome-Wide Identification of the Transient Receptor Potential Channel Family in Nile Tilapia and Expression Analysis in Response to Cold Stress
Source: Animals (Basel). 2025 Dec 18;15(24):3645. doi: 10.3390/ani15243645 (PMC12729289; doi:10.3390/ani15243645)
Supplement: Supplementary file 1 [file animals-15-03645-s001.zip › Supplementary Table S1.pdf]

Supplementary Table S1 Primer sequences used in this study

Primer name	Sequences（from 5' to 3'）	Purpose	Primer accession	Annealing temperature (℃)	Amplicon size (bp)
TRPC5-RT-F	TACTATGAAACCAAGGCATCAGAGG	RT-PCR	Designed in this study	60.4	481
TRPC5-RT-R	ACTCCCGCAGGTTTTCACATTTG			62.11	
β-actin-RT-F	GGCATCACACCTTCTACAACGA		Used in previous study [1]	60.61	332
β-actin-RT-R	ACGCTCTGTCAGGATCTTCA			58.45	
TRPC5-ISH-F	GGACTCAGCAACGTGACCAGAAG	In situ hybridization	Designed in this study	63.48	539
TRPC5-ISH-R	TCCATTCTCGGGTGTGCTGC			62.80	
β-actin-Q-F	GACAACGGATCCGGTATGTGC	RT-qPCR	Designed in this study	61.40	141
β-actin-Q-R	CTCATCACCAACGTAGCTGTC			58.74	
TRPC5-Q-F	CAGGTTCCTTCTCTGATGATGG		Designed in this study	58.19	129
TRPC5-Q-R	TATGGTGACCTTCCGCTGG			59.10	
TRPM7-Q-F	AGGTGTAGCAAAACATGTGGGAG		Designed in this study	61.06	137
TRPM7-Q-R	GAGCAATAATGTCTCTGCCGATG			59.81	

References：

[1] Wei L, Tang Y, Zeng X, Li Y, Zhang S, Deng L, Wang L, Wang D. The transcription factor Sox30 is involved in Nile tilapia spermatogenesis. J Genet Genomics. 2022, 49(7): 666-676.
