# Supplementary material for: Genome-Wide Identification of the Transient Receptor Potential Channel Family in Nile Tilapia and Expression Analysis in Response to Cold Stress
Source: Animals (Basel). 2025 Dec 18;15(24):3645. doi: 10.3390/ani15243645 (PMC12729289; doi:10.3390/ani15243645)
Supplement: Supplementary file 1 [file animals-15-03645-s001.zip › Supplementary Table S2.pdf]

Supplementary Table S2 Molecular characteristics of TRP proteins in the Nile tilapia

Protein name	Accession Number (NCBI)	Instability Index	GRAVY	Secondary structure			Subcellular localization
				Alpha helix Content	Beta turn Content	Random coil Content	
TRPA1	XP_025766241.1	40.95	0.006	49.64%	7.87%	32.83%	endomembrane system
TRPC1	XP_003439260.1	42.7	-0.072	57.74%	3.90%	32.20%	endomembrane system
TRPC2	XP_025766801.1	46.03	-0.341	57.92%	3.37%	33.37%	plasma membrane
TRPC4a	XP_005472340.1	48.68	-0.256	55.68%	3.20%	34.81%	endomembrane system
TRPC4b	XP_013129815.1	41.67	-0.163	59.81%	3.29%	30.90%	endomembrane system
TRPC5	XP_003455059.3	55.15	-0.246	50.32%	4.67%	35.84%	plasma membrane
TRPC6a	XP_003446791.2	42.52	-0.033	57.00%	2.84%	34.02%	organelle membrane
TRPC6b	XP_025753927.1	45.09	-0.081	59.67%	2.93%	30.83%	organelle membrane
TRPC7	XP_003444730.1	47.98	-0.276	49.11%	3.47%	40.58%	endomembrane system
TRPM1a	XP_025764567.1	48.29	-0.4	42.89%	5.01%	39.02%	endomembrane system
TRPM1b	XP_019216783.1	42.1	-0.37	47.86%	2.63%	40.04%	endomembrane system
TRPM2	XP_005453114.1	43.58	-0.195	48.16%	2.52%	38.69%	plasma membrane
TRPM3	XP_025752804.1	48.97	-0.294	42.86%	4.06%	42.25%	endomembrane system
TRPM4a	XP_005448371.1	44.45	-0.195	48.93%	3.48%	36.98%	plasma membrane
TRPM4b1	XP_019218187.1	43.83	-0.178	48.89%	3.49%	37.38%	plasma membrane
TRPM4b2	XP_019218227.1	43.68	-0.191	49.57%	3.16%	36.54%	plasma membrane
TRPM4b3	XP_019218186.1	44	-0.188	47.72%	3.46%	38.27%	plasma membrane
TRPM5	XP_005455786.1	39.24	-0.176	48.70%	3.16%	37.20%	plasma membrane
TRPM6	XP_005473206.1	51.73	-0.362	44.56%	4.63%	41.29%	plasma membrane
TRPM7	XP_025763107.1	50.41	-0.288	41.91%	4.48%	42.95%	plasma membrane
TRPV1	XP_013120120.1	43.86	-0.23	48.47%	3.94%	37.40%	plasma membrane
TRPV4	XP_003451782.1	45.52	-0.16	47.13%	3.56%	39.77%	endomembrane system
TRPV6	XP_019219982.1	43.55	0.127	53.16%	4.08%	32.91%	endomembrane system
TRPP1a	XP_013119868.1	46.49	-0.145	23.25%	4.71%	44.89%	plasma membrane
TRPP1b	XP_019217617.2	45.87	0.009	25.48%	4.11%	44.66%	plasma membrane
TRPP2	XP_005457010.1	52.17	-0.345	54.16%	2.77%	35.07%	plasma membrane
TRPP3	XP_003441269.1	30.27	-0.075	58.17%	2.75%	29.54%	plasma membrane
TRPML1a	XP_005470236.1	37.78	0.128	38.92%	4.57%	36.21%	plasma membrane
TRPML1b	XP_003443211.1	34.67	0.23	43.78%	4.73%	31.17%	endomembrane system
TRPML2	XP_025754989.1	35.01	0.131	45.63%	4.56%	28.57%	endomembrane system
TRPML3a	XP_019203764.1	33.03	0.057	42.67%	4.21%	32.42%	plasma membrane
TRPML3b	XP_005457189.1	35.98	0.028	42.93%	3.58%	31.84%	plasma membrane
