# Supplementary material for: Genome-Wide Identification of the Transient Receptor Potential Channel Family in Nile Tilapia and Expression Analysis in Response to Cold Stress
Source: Animals (Basel). 2025 Dec 18;15(24):3645. doi: 10.3390/ani15243645 (PMC12729289; doi:10.3390/ani15243645)
Supplement: Supplementary file 1 [file animals-15-03645-s001.zip › Supplementary Table S3.pdf]

Supplementary Table S3 Inventory of the TRP family in several representative animals

Fruit fly (*Drosophila melanogaster*, Dm)		Mouse (*Mus musculus*, Mm)		Human (*Homo sapiens*, Hs)		Spotted gar (*Lepisosteus oculatus*, Lo)		Medaka (*Oryzias latipes*, Ol)		Zebrafish (*Danio rerio*, Dr)		Channel catfish (*Ictalurus punctatus*, Ip)		Common carp (*Cyprinus carpio*, Cc)		Rainbow trout (*Oncorhynchus mykiss*, Om)	
Gene name	NCBI accession number	Gene name	NCBI accession number	Gene name	NCBI accession number	Gene name	NCBI accession number	Gene name	NCBI accession number	Gene name	NCBI accession number	Gene name	NCBI accession number	Gene name	NCBI accession number	Gene name	NCBI accession number
DmTRPA1	NP_001261602.1	*MmTRPA1*	NP_001335217.1	*HsTRPA1*	XP_011515926.1	*LoTRPA1*	XP_015208897.1	*OlTRPA1b*	XP_023806150.1	*DrTRPA1a*	NP_001007066.1	*IpTRPA1a*	XP_017351760.1	*CcTRPA1*	XP_042608085.1	*OmTRRPA1a*	XP_036841885.1
DmTRPA-pyx	NP_612015.1	*MmTRPC1*	NP_001298052.2	*HsTRPC1*	NP_001238774.1	*LoTRPC1*	XP_006637772.1	*OlTRPC1*	XP_020568461.1	*DrTRPA1b*	NP_001007067.1	*IpTRPA1b*	XP_017309091.1	*CcTRPA1-like1*	XP_042630489.1	*OmTRPA1b1*	XP_036822207.1
DmTRPA-wtrw	NP_731194.1	*MmTRPC2*	NP_001103367.1	*HsTRPC2*	pseudogene	*LoTRPC2*	XP_015196659.1	*OlTRPC2-like*	XP_023818592.1	*DrTRPC1*	NP_001185590.1	*IpTRPC1*	XP_017347310.1	*CcTRPA1-like2*	XP_042570143.1	*OmTRPA1b2*	XP_036790569.1
DmTRPA-pain	NP_001261176.1	*MmTRPC3*	NP_062383.2	*HsTRPC3*	NP_003296.1	*LoTRPC3*	XP_015199496.1	*OlTRPC4a*	XP_004076346.1	*DrTRPC2a*	XP_017206723.1	*IpTRPC2*	XP_017347309.1	*CcTRPC1-like1*	XP_042607670.1	*OmTRPA1-like*	XP_036801568.1
DmTRP	NP_476768.1	*MmTRPC4*	NP_058680.1	*HsTRPC4*	NP_057263.1	*LoTRPC4*	XP_006628285.1	*OlTRPC4b*	XP_004075586.1	*DrTRPC2b*	NP_001025337.2	*IpTRPC3*	XP_017340202.1	*CcTRPC1-like2*	XP_042570017.1	*OmTRPC1*	NP_001171982.1
DmTRP-like	NP_476895.1	*MmTRPC5*	NP_033454.1	*HsTRPC5*	NP_036603.1	*LoTRPC5*	XP_015206809.1	*OlTRPC5*	XP_023804759.1	*DrTRPC3*	NP_001276813.2	*IpTRPC4a*	XP_017343908.1	*CcTRPC2*	XP_042567423.1	*OmTRPC2-like1*	XP_036793250.1
DmTRP-gamma	NP_001137830.2	*MmTRPC6*	NP_038866.2	*HsTRPC6*	XP_011541270.1	*LoTRPC6*	XP_006628127.2	*OlTRPC6a*	XP_004076431.2	*DrTRPC4a*	NP_001276811.1	*IpTRPC4b*	XP_017346347.1	*CcTRPC2-like1*	XP_042627696.1	*OmTRPC2-like2*	XP_036817775.1
DmTRPM	NP_001137672.2	*MmTRPC7*	NP_036165.1	*HsTRPC7*	NP_065122.1	*LoTRPC7*	XP_015205153.1	*OlTRPC6b*	XP_023817144.1	*DrTRPC4b*	NP_001276810.1	*IpTRPC5*	XP_017317555.1	*CcTRPC2-like2*	XP_042596015.1	*OmTRPC3*	XP_036826284.1
DmTRPN-nompC	NP_001245891.1	*MmTRPM1*	NP_001034193.2	*HsTRPM1*	NP_001238949.1	*LoTRPM1*	XP_006629107.1	*OlTRPC7-like*	XP_020562217.2	*DrTRPC5a*	NP_001314676.2	*IpTRPC6a*	XP_017348488.1	*CcTRPC3*	XP_042593897.1	*OmTRPC3-like*	XP_021417130.2
DmTRPP2	NP_609561.2	*MmTRPM2*	NP_612174.2	*HsTRPM2*	XP_011528038.1	*LoTRPM2*	XP_015214648.1	*OlTRPM1a*	XP_023811560.1	*DrTRPC5b*	XP_009296662.2	*IpTRPC6b*	XP_017320832.1	*CcTRPC3-like*	XP_042626617.1	*OmTRPC4a*	XP_036823620.1
DmTRPV-iav	NP_572353.1	*MmTRPM3*	XP_006527017.1	*HsTRPM3*	XP_011517340.1	*LoTRPM3*	XP_015222955.1	*OlTRPM1b.1*	XP_023811561.1	*DrTRPC6a*	NP_001025453.1	*IpTRPC7a*	XP_017345016.1	*CcTRPC4*	XP_042595719.1	*OmTRPC4b*	XP_036821404.1
DmTRPV-nan	NP_648696.2	*MmTRPM4*	NP_780339.2	*HsTRPM4*	NP_001182156.1	*LoTRPM4*	XP_015208857.1	*OlTRPM1b.2*	XP_011485404.1	*DrTRPC6b*	XP_002665445.2	*IpTRPC7b*	XP_017340141.1	*CcTRPC4-like1*	XP_042627343.1	*OmTRPC4-like*	XP_021438058.2
DmTRPML	NP_649145.1	*MmTRPM5*	NP_064673.2	*HsTRPM5*	NP_055370.1	*LoTRPM5*	XP_015193971.1	*OlTRPM2*	XP_023806259.1	*DrTRPC7a*	NP_001276808.1	*IpTRPM1*	XP_047015731.1	*CcTRPC4-like2*	XP_042588499.1	*OmTRPC5a*	XP_021474547.2
		MmTRPM6	NP_700466.1	*HsTRPM6*	NP_060132.3	*LoTRPM6*	XP_015222636.1	*OlTRPM3*	XP_020561683.1	*DrTRPC7b*	NP_001276815.1	*IpTRPM2*	XP_017324684.1	*CcTRPC4-like3*	XP_042621023.1	*OmTRPC5b1*	XP_036823932.1
		MmTRPM7	NP_067425.2	*HsTRPM7*	NP_060142.3	*LoTRPM7*	XP_006628750.2	*OlTRPM4a*	XP_023813623.1	*DrTRPM1a*	NP_001074147.2	*IpTRPM3*	XP_047005918.1	*CcTRPC5*	XP_042587463.1	*OmTRPC5b2*	XP_036814235.1
		MmTRPM8	NP_599013.1	*HsTRPM8*	XP_016860380.1	*LoTRPN1*	XP_006635908.2	*OlTRPM4b*	XP_011486369.1	*DrTRPM1b*	XP_021326196.1	*IpTRPM4a*	XP_017350642.1	*CcTRPC5-like1*	XP_042572799.1	*OmTRPC5-like*	XP_036845130.1
		MmTRPP1	NP_038658.2	*HsTRPP1*	NP_000287.4	*LoTRPP1a*	XP_015215450.1	*OlTRPM5*	XP_023808229.1	*DrTRPM2*	XP_009303267.1	*IpTRPM4b*	XP_017339465.1	*CcTRPC5-like2*	XP_042567099.1	*OmTRPC6a*	XP_021472999.1
		MmTRPP2	XP_006534878.1	*HsTRPP2*	NP_000288.1	*LoTRPP1b*	XP_015211579.1	*OlTRPM6*	XP_020561734.1	*DrTRPM3*	XP_021332143.1	*IpTRPM5*	XP_017313908.1	*CcTRPC5-like3*	XP_042604034.1	*OmTRPC6b*	XP_036821591.1
		MmTRPP3	NP_852087.2	*HsTRPP3*	NP_001240766.1	*LoTRPP2*	XP_006630139.1	*OlTRPM7*	XP_020555660.1	*DrTRPM4a*	NP_001275744.2	*IpTRPM6*	XP_017307822.1	*CcTRPC6a*	XP_042566797.1	*OmTRPC7a*	XP_036798664.1
		MmTRPP5	NP_001156476.1	*HsTRPP5*	NP_001245377.1	*LoTRPP3*	XP_015203008.1	*OlTRPP1a*	NP_001129985.1	*DrTRPM4b1*	XP_009304689.1	*IpTRPM7*	XP_047010472.1	*CcTRPC6b*	XP_042599670.1	*OmTRPC7b*	XP_021474157.1
		MmTRPV1	NP_001001445.1	*HsTRPV1*	NP_061197.4	*LoTRPP5*	XP_015205289.1	*OlTRPP1b*	XP_011488440.2	*DrTRPM4b2*	NP_001269082.1	*IpTRPN1*	XP_047006463.1	*CcTRPC7-like1*	XP_042587923.1	*OmTRPC7-like*	XP_036826336.1
		MmTRPV2	NP_001369418.1	*HsTRPV2*	NP_057197.2	*LoTRPV1*	XP_006641065.1	*OlTRPP2a*	XP_011480856.1	*DrTRPM4b3*	NP_001314961.1	*IpTRPP1a*	XP_047007109.1	*CcTRPC7-like2*	XP_042593647.1	*OmTRPM1a*	XP_036790456.1

		MmTRPV3	NP_659567.2	*HsTRPV3*	NP_001245134.1	*LoTRPV4*	XP_006640397.1	*OlTRPP2b*	NP_001129990.1	*DrTRPM5*	NP_001121711.1	*IpTRPP1b*	XP_047015522.1	*CcTRPC7-like3*	XP_042626464.1	*OmTRPM1b1*	XP_036819593.1
		MmTRPV4	NP_071300.2	*HsTRPV4*	XP_011536932.2	*LoTRPV6*	XP_006642484.1	*OlTRPP3*	NP_001239172.1	*DrTRPM6*	XP_021331654.1	*IpTRPP2*	XP_017318790.1	*CcTRPM1-like1*	XP_042583515.1	*OmTRPM1b2*	XP_036819589.1
		MmTRPV5	NP_001007573.1	*HsTRPV5*	NP_062815.3	*LoTRPMLl1*	XP_015204501.1	*OlTRPV1a*	XP_011482044.1	*DrTRPM7*	NP_001025232.1	*IpTRPP3*	XP_017319090.1	*CcTRPM1-like2*	XP_042615939.1	*OmTRPM1-like1*	XP_036820450.1
		MmTRPV6	NP_071858.3	*HsTRPV6*	NP_061116.5	*LoTRPML2*	XP_015210871.1	*OlTRPV1b*	XP_011480561.1	*DrTRPN1*	NP_899192.1	*IpTRPV1a*	XP_017315455.1	*CcTRPM1-like3*	XP_042571503.1	*OmTRPM1-like2*	XP_036837542.1
		MmTRPMLl1	NP_444407.1	*HsTRPML1*	NP_065394.1	*LoTRPML3*	XP_015210611.1	*OlTRPV4*	XP_020561608.1	*DrTRPP1a*	XP_021333081.1	*IpTRPV1b*	XP_017315459.1	*CcTRPM2*	XP_042586376.1	*OmTRPM2*	XP_036815342.1
		MmTRPML2	NP_001005846.1	*HsTRPML2*	NP_001317576.1			*OlTRPV6*	XP_004078007.1	*DrTRPP1b*	XP_021335980.1	*IpTRPV4*	XP_017307076.1	*CcTRPM2-like1*	XP_042571934.1	*OmTRPM2-like*	XP_036829842.1
		MmTRPML3	NP_598921.1	*HsTRPML3*	NP_001240622.1			*OlTRPML1a*	XP_011475428.1	*DrTRPP2*	NP_001002310.1	*IpTRPV6*	XP_017334756.1	*CcTRPM2-like2*	XP_042620293.1	*OmTRPM3a*	XP_021476775.2
								OlTRPML1b	XP_004072026.2	*DrTRPP3*	XP_695404.3	*IpTRPML1a*	XP_017313585.1	*CcTRPM3*	XP_042579819.1	*OmTRPM3b*	XP_036835448.1
								OlTRPML2	XP_004067779.1	*DrTRPV1*	NP_001119871.1	*IpTRPML1b*	XP_017337098.1	*CcTRPM3-like*	XP_042610697.1	*OmTRPM4a*	XP_036793842.1
								OlTRPML3a	XP_020566850.1	*DrTRPV4*	NP_001036195.1	*IpTRPML2*	XP_017333717.1	*CcTRPM4*	XP_042579807.1	*OmTRPM4b1*	XP_036793843.1
								OlTRPML3b	XP_004068128.1	*DrTRPV6*	NP_001001849.1	*IpTRPML3a*	XP_017328587.1	*CcTRPM5*	XP_042616583.1	*OmTRPM4b2*	XP_021421236.2
										DrTRPML1a	NP_001315094.1	*IpTRPML3b*	XP_017343616.1	*CcTRPM5-like1*	XP_042584108.1	*OmTRPM4b3*	XP_036794715.1
										DrTRPML1b	NP_001373555.1			*CcTRPM6-like2*	XP_042610571.1	*OmTRPM5a*	XP_036837354.1
										DrTRPML2	NP_957442.1			*CcTRPM7*	XP_042599562.1	*OmTRPM5b*	XP_036819530.1
										DrTRPML3a	XP_021322333.1			*CcTRPM7-like1*	XP_042631784.1	*OmTRPM6*	XP_021461144.2
										DrTRPML3b	XP_021325745.1			*CcTRPM7-like2*	XP_042579797.1	*OmTRPM7a*	XP_036836924.1
														CcTRPM7-like3	XP_042599565.1	*OmTRPM7b1*	XP_036819961.1
														CcTRPN1	XP_042601938.1	*OmTRPM7b2*	XP_036792206.1
														CcTRPP1	XP_042623498.1	*OmTRPN1*	XP_036827340.1
														CcTRPP1-like1	XP_042619433.1	*OmTRPP1*	XP_036828951.1

														CcTRPP1-like2	XP_042573283.1	*OmTRPP2a*	XP_036810100.1
														CcTRPP2	XP_042617852.1	*OmTRPP2b*	XP_036789594.1
														CcTRPP3	XP_018961396.1	*OmTRPP3*	XP_021462796.2
														CcTRPP5	XP_018961395.2	*OmTRPV1*	XP_021473699.2
														CcTRPV1-like1	XP_042613261.1	*OmTRPV4a*	XP_036791455.1
														CcTRPV1-like2	XP_042613304.1	*OmTRPV4b*	XP_021461081.2
														CcTRPV1-like3	XP_042580385.1	*OmTRPV5*	XP_021464062.1
														CcTRPV4-like1	XP_042579542.1	*OmTRPV6*	XP_036824702.1
														CcTRPV4-like2	XP_042606953.1	*OmTRPV6-like*	XP_036819747.1
														CcTRPV5-like1	XP_042596552.1	*OmTRPML1a*	XP_021412901.1
														CcTRPV5-like2	XP_042628045.1	*OmTRPMLl1b1*	XP_036805573.1
														CcTRPML1a	XP_042573107.1	*OmTRPML1b2*	XP_021480681.2
														CcTRPML1b	XP_042577048.1	*OmTRPMLl1b3*	XP_021413814.2
														CcTRPML1-like	XP_042616858.1	*OmTRPML2*	XP_021455642.1
														CcTRPML2-like1	XP_042605328.1	*OmTRPML3a*	XP_021444786.1
														CcTRPML2-like2	XP_042567862.1	*OmTRPML3b*	XP_021460258.2
														CcTRPML3	XP_042606989.1		
														CcTRPML3-like	XP_042579227.1		

Note: The red color indicates newly-identified genes.
